# Low-dose esketamine for postoperative fatigue after gynecological laparoscopic surgery: a randomized controlled trial

**DOI:** 10.1186/s12871-026-04051-z

**Published:** 2026-06-26

**Authors:** Ge Sun, Wentao Lin, Yujin Peng, Zhuming Liang, Haiming Liao, Jun Zhou, Fuhu Song

**Affiliations:** Department of Anesthesiology, The Third Affiliated Hospital, Southern Medical University, 183 Zhongshan Avenue West, Tianhe District, Guangzhou, Guangdong China

**Keywords:** Esketamine, Postoperative fatigue, Gynecological laparoscopic surgery, Postoperative recovery quality

## Abstract

**Background:**

Postoperative fatigue is a prevalent complication delaying early recovery after gynecological laparoscopic surgery, with no proven effective interventions. To investigate the effect of continuous intraoperative low-dose esketamine on postoperative fatigue in patients undergoing gynecological laparoscopic surgery.

**Methods:**

This single-center, double-blind, randomized placebo-controlled trial enrolled 116 elective gynecological laparoscopic surgery patients, randomly assigned 1:1 to the esketamine group (0.2 mg·kg⁻¹·h⁻¹ esketamine infusion during surgery, *n* = 58) or control group (equal volume normal saline, *n* = 58). The primary outcome was the incidence of postoperative fatigue on postoperative day 1 (POD1, Chalder Fatigue Scale score ≥ 4). Secondary outcomes included Chalder Fatigue Scale scores, 15-item Quality of Recovery (QoR-15) scores, Visual Analogue Scale (VAS) pain scores, perioperative drug consumption, and safety events.

**Results:**

In the primary intention-to-treat analysis, the incidence of POD1 postoperative fatigue was 23.3% (14/60) in the esketamine group and 40.0% (24/60) in the control group (risk difference − 16.7%, OR = 0.46, 95% CI 0.21–1.01, *P* = 0.050). Esketamine group had significantly lower POD1 fatigue scores, better recovery quality and pain control on POD1 and POD3, reduced intraoperative sufentanil and ephedrine consumption, (all *P* < 0.05). No significant differences in adverse events were observed between groups (all *P* > 0.05).

**Conclusion:**

Intraoperative low-dose esketamine shows a favorable effect on reducing postoperative fatigue, and significantly improves recovery quality and analgesia, without increasing adverse events in patients undergoing gynecological laparoscopic surgery. The primary ITT analysis shows a borderline significant reduction in fatigue incidence, which is supported by a statistically significant result in the per-protocol sensitivity analysis.

**Trial registration:**

This study was prospectively registered at the Chinese Clinical Trial Registry (ChiCTR, https://www.chictr.org.cn/showproj.html?proj=290957), with the clinical trial registration number ChiCTR2500112382, and the registration date is November 13, 2025.

## Introduction

Gynecological laparoscopic surgery offers advantages of minimal trauma and rapid recovery. It has become a common procedure for the diagnosis and treatment of gynecological diseases [1]. Postoperative fatigue syndrome is a common complication of this type of surgery and significantly impairs early postoperative recovery [2]. Postoperative fatigue is characterized by persistent physical and mental exhaustion, decreased activity tolerance, and impaired cognitive function. Its occurrence is closely associated with multiple factors, including surgical trauma-induced stress, inflammatory response, sleep disturbance, and pain [3]. The condition not only affects patients’ quality of life but also prolongs hospital stays, increases the risk of readmission, and delays return to work and social activities [4]. However, effective interventions for postoperative fatigue are currently lacking [3]. 

In this context, esketamine may offer a new therapeutic approach. Esketamine is the S (+) - enantiomer of racemic ketamine, a non-competitive N-methyl-D-aspartate (NMDA) receptor antagonist. It has 3- to 4-fold higher binding affinity for the NMDA receptor than the R (−) - enantiomer, resulting in approximately twice the analgesic potency of racemic ketamine and a more favorable neuropsychiatric adverse effect profile [5, 6]. By blocking the NMDA receptor, esketamine inhibits glutamatergic neurotransmission, thereby exerting anesthetic, analgesic, and rapid antidepressant effect [7, 8]. These properties provide a theoretical basis for its use in perioperative management to improve postoperative outcomes.

Perioperative administration of esketamine has been shown to significantly reduce postoperative fatigue scores, as demonstrated in various surgical procedures (including laparoscopic radical gastrectomy and total hysterectomy) and with different administration regimens (continuous infusion and single preoperative intravenous injection) [9–12]. These findings sµggest that esketamine has potential therapeutic value for postoperative fatigue. In recent years, several clinical studies have confirmed that perioperative use of esketamine also has positive effects on opioid consumption, quality of recovery, and sleep quality in patients undergoing different types of surgery [13–15]. Esketamine has also shown advantages in reducing nausea and vomiting and decreasing the risk of postoperative delirium [16–20]. Common neurological adverse reactions associated with esketamine mainly manifest as transient psychotic-like symptoms such as dizziness, diplopia, euphoria, and hallucinations that appear shortly after administration and usually resolve spontaneously [21, 22]. 

However, high-quality prospective randomized controlled evidence regarding whether continuous intraoperative infusion of low-dose esketamine effectively reduces the incidence of postoperative fatigue after gynecological laparoscopic surgery remains lacking. Existing studies have predominantly focused on major abdominal surgery [23], and research specifically targeting the gynecological laparoscopic surgery population is still limited. Gynecological laparoscopic surgery has unique clinical characteristics. Patients are predominantly female, and rapid, high-quality recovery is particularly important given their multiple roles in family and society [24]. The procedure itself may involve reproductive organ resection, which can have complex psychological and emotional impacts [25]. In addition, the physiological disturbances caused by pneumoperitoneum and special positioning differ from those associated with other surgical procedures [26]. 

Therefore, this study conducted a randomized, double-blind, controlled trial to investigate the effect of continuous infusion of low-dose esketamine on postoperative fatigue in patients undergoing gynecological laparoscopic surgery. We pre-specified the primary hypothesis that intraoperative continuous infusion of low-dose esketamine would significantly reduce the incidence of postoperative fatigue on postoperative day 1 in patients undergoing gynecological laparoscopic surgery, compared with normal saline placebo. We evaluated its clinical value from multiple dimensions, including the incidence of postoperative fatigue, quality of recovery, pain control, and drµg consumption, aiming to provide new evidence for optimizing multimodal analgesia strategies and promoting early postoperative recovery in this surgical setting.

## Methods

### Study design and approvals

This randomized controlled trial was conducted and reported in strict accordance with the Consolidated Standards of Reporting Trials (CONSORT) 2010 statement guidelines. This was a single-center, double-blind, parallel-group, randomized, placebo-controlled trial with a 1:1 allocation ratio between the esketamine intervention group and the normal saline control group. The study was conducted in accordance with the Declaration of Helsinki and guidelines for good clinical practice. The protocol was approved by the Ethics Committee of the Third Affiliated Hospital of Southern Medical University (approval No. 2025-ER-033) and registered at the Chinese Clinical Trial Registry (ChiCTR2500112382).

No important changes to the trial protocol, including eligibility criteria, intervention regimens, outcome measures, and statistical analysis plans, were made after the trial commencement and patient enrollment. No major protocol deviations occurred during the study implementation.

All outcome analyses reported in this section were fully pre-specified in the study protocol and clinical trial registration before trial commencement. No post-hoc exploratory analyses, subgroup analyses, or adjusted multivariable analyses were performed in this study.

### Patients

Patients undergoing gynecological laparoscopic surgery at the Third Affiliated Hospital of Southern Medical University were eligible for participation. Inclusion criteria were age 18 ~ 65 years, American Society of Anesthesiologists (ASA) physical status I or II, and scheduled for gynecological laparoscopic surgery. Exclusion criteria were ASA physical status III or higher, unstable angina or myocardial infarction within the previous 3 months, allergy to study or anaesthetic drµgs, untreated or uncontrolled hypertension (resting blood pressure > 180/100 mmHg), congestive heart failure, severe hepatic or renal dysfunction, elevated intracranial pressure or central nervous system disease, body mass index < 18 or > 30 kg/m², and participation in another clinical trial within the previous month. Written informed consent was obtained from all patients. This study was conducted at the Department of Anesthesiology, Department of Gynecology, and Post-Anesthesia Care Unit (PACU) of the Third Affiliated Hospital of Southern Medical University (Guangzhou, Guangdong, China). All participant screening, informed consent signing, surgical intervention, data collection, and follow-up were completed at this single study center.

### Randomisation, study drµg and blinding

Patients were randomly assigned in a 1:1 ratio to either the esketamine group or the control group. The randomization sequence was generated by an independent statistician using a computer-generated random number table, with a block size of 4 to ensure balanced allocation between groups. Stratification by surgical type was not performed, however, baseline characteristics including surgical type were well balanced between the two groups (Table 1), confirming effective randomization. The randomization sequence was concealed in sequentially numbered, sealed, opaque envelopes. One hour before surgery, the envelope was opened by an independent anesthesia nurse who was not involved in intraoperative anesthesia management, patient follow-up, or outcome assessment, to ensure the randomness and unpredictability of the allocation process.

**Table 1:** Baseline characteristics

Characteristic	Group E (*n*=58)	Group C (*n*=58)	*P* value
General Information	41.0(29.8,50)	41.1(32,50)	0.682
Weight, mean ± SD, kg	57.2±8.2	57.5±7.3	0.852
Height, mean ± SD, cm	159.1±4.7	158.2±5.7	0.378
BMI, mean ± SD, kg/m²	22.6±2.7	23.0±2.6	0.426
ASA physical status II, n (%)	60(100)	60(100)	—
Previous abdominal surgery, n (%)	23, (38.3%)	24, (40%)	1
Preoperative haemoglobin, median (IQR), g/L	118.0(111.8,127.5)	122.5(104,130)	0.862
Comorbidities, n (%)			
Hypertension	7, (12%)	3, (5%)	0.32
Diabetes mellitus	2, (3%)	2, (5%)	1
Anaemia	14, (24%)	16, (28%)	0.83
Thyroid dysfunction	2, (3%)	1, (2%)	1
Preoperative scores, mean ± SD			
Preoperative Chalder Fatigue Scale score	1.17±1.7	0.75±1.22	0.13
Preoperative Pittsburgh Sleep Quality Index score	4.35±2.8	5.0±3.3	0.27
Type of surgery, n (%)			
Ovarian lesion resection	31, (52%)	28, (47%)	0.715
Uterine lesion resection	11, (18%)	16, (27%)	0.382
Tubal surgery	46, (77%)	40, (67%)	0.311
Total hysterectomy	23, (38%)	17, (28%)	0.333
Hysteroscopic surgery	16, (27%)	23. (38%)	0.242
Other	0, (0%)	2, (3%)	0.496

All data in this table are presented based on the intention-to-treat (ITT) full analysis set (FAS), with 60 patients in the esketamine group (Group E) and 60 patients in the normal saline control group (Group C). Data are presented as n (%), mean ± standard deviation (SD), or median (interquartile range, IQR). Group E received continuous intraoperative infusion of 0.2 mg·kg⁻¹·h⁻¹ esketamine; Group C received equal volume of normal saline. ASA: American Society of Anesthesiologists; BMI: body mass index. A *P* value < 0.05 was considered statistically significant. There were no statistically significant differences in all baseline characteristics between the two groups (all *P* > 0.05)

To maintain double-blinding, patients, attending anesthesiologists, intraoperative care providers, postoperative outcome assessors, and data analysts were all blinded to group allocation throµghout the study period. Only the independent anesthesia nurse responsible for study drµg preparation had access to the group allocation information. The study drµg (esketamine or normal saline) was prepared by the independent anesthesia nurse in identical, unlabeled syringes with equal volume, and delivered to the attending anesthesiologist before surgery. Patients in the esketamine group received a continuous intravenous infusion of esketamine at 0.2 mg·kg⁻¹·h⁻¹ from the start of surgery until the end of the procedure, while patients in the control group received an equal volume of normal saline via the same infusion regimen.

The allocation information was stored in sealed opaque envelopes for emergency unblinding. Emergency unblinding would only be performed in the event of a serious adverse event that required immediate knowledge of group allocation for clinical management, and the unblinding event would be fully documented and reported. The allocation concealment was maintained until the completion of all statistical analyses and the final draft of the manuscript.

### Anesthesia and postoperative pain management

Surgery and anesthesia followed standard procedures at the Third Affiliated Hospital of Southern Medical University. Anesthesia was induced with remimazolam 0.25 mg/kg, etomidate 0.3 mg/kg, sufentanil 0.5 µg/kg, and cisatracurium 0.2 mg/kg. After tracheal intubation, mechanical ventilation was initiated to maintain end-tidal carbon dioxide pressure at 35 ~ 45 mmHg. Anesthesia was maintained with 1.0 minimum alveolar concentration (MAC) of sevoflurane, combined with etomidate 0.2 ~ 0.3 mg·kg⁻¹·h⁻¹and remifentanil 0.2 ~ 0.4 µg·kg⁻¹·min⁻¹, with additional cisatracurium administered as needed to maintain neuromuscular blockade.

Low-dose continuous etomidate infusion was used for short-term maintenance of anesthesia in this study. The total duration of etomidate infusion was < 4 h in all patients, with a total dose < 0.5 mg/kg. Accumulating evidence demonstrates that short-term low-dose etomidate infusion does not cause clinically significant adrenal suppression or increase adverse event risk in elective surgery [27]. 

The remifentanil infusion rate was adjusted according to heart rate and blood pressure, and vasoactive agents were used when necessary to maintain blood pressure within 20% of baseline values. All anesthesiologists followed the same standardized hemodynamic protocol, with identical blood pressure targets (maintain within 20% of baseline) for both groups. The difference in ephedrine consumption reflects the hemodynamic effect of esketamine, not differences in clinical practice.

All patients received ultrasound-guided bilateral transversus abdominis plane block (0.3% ropivacaine, 20 ml per side) after surgery. Postoperative pain was managed according to pain intensity measured using the Visual Analogue Scale (VAS, 0 ~ 10, where 0 = no pain and 10 = worst possible pain). If the VAS score was ≥ 4, tramadol 100 mg was administered intravenously as rescue analgesia. If SpO₂ decreased to < 95%, FiO₂ was increased, and mask ventilation was provided if necessary. If nausea or vomiting occurred, ondansetron 2 mg was administered intravenously.

### Outcomes

The primary outcome was the incidence of postoperative fatigue on postoperative day 1, defined as a Chalder Fatigue Scale score ≥ 4. This cutoff has been widely validated and adopted in surgical populations to identify clinically meaningful postoperative fatigue [28]. Secondary outcomes included Chalder Fatigue Scale scores, 15-item Quality of Recovery (QoR-15) scores [29], and Visual Analogue Scale (VAS) pain scores on postoperative days 1 and 3; intraoperative consumption of sufentanil and ephedrine; rescue analgesia rate; and the incidence of adverse reactions, including nausea and vomiting, hypoxaemia, hypertension, postoperative agitation, and delayed emergence. All primary and secondary outcome measures, including their assessment tools, time points, and diagnostic criteria, were fully pre-specified in the study protocol and clinical trial registration before trial commencement and patient enrollment. No changes were made to the outcome measures after the trial started.

### Sample size

Sample size was calculated based on the results of a preliminary study. In the preliminary study, the incidence of postoperative fatigue on day 1 was 53.8% in the esketamine group and 84.6% in the control group. With a two-sided α of 0.05 and a β of 0.1 (90% statistical power), 46 patients were required in each group for the per-protocol analysis. To account for a 20% dropout rate, 58 patients per group were targeted for the per-protocol set, and a total of 120 patients (60 per group) were randomized to allow for exclusions and post-randomization attrition.

The initial trial registration planned for 53 patients per group based on 80% statistical power. The sample size was subsequently increased to 58 per group to achieve 90% power before enrollment began, and the trial registry record was updated accordingly. A total of 120 patients (60 per group) were randomized to accommodate exclusions and attrition.

No interim analysis was pre-specified in the study protocol, and no stopping guidelines for early termination of the trial were set. The trial was completed as scheduled after the pre-specified sample size was fully enrolled.

### Statistical analysis

Statistical analyses were performed using SPSS for Windows version 26.0. Normality of continuous variables was assessed using the Shapiro-Wilk test. Normally distributed data are presented as mean ± standard deviation and were compared using independent samples t-test. Non-normally distributed data are presented as median (interquartile range) and were compared using Mann-Whitney U test. Categorical variables are presented as numbers (percentages) and were compared using chi-square test or Fisher’s exact test as appropriate. All statistical tests were two-sided, and a *P* value < 0.05 was considered statistically significant.

This study adopted a randomized controlled design, and a total of 120 participants were enrolled and randomly allocated in a 1:1 ratio to two groups (60 participants per group). During postoperative follow-up, a total of 4 participants failed to complete the follow-up assessments on postoperative day 1 (POD1) and postoperative day 3 (POD3), including 2 participants in the esketamine group (1 excluded from follow-up analysis due to anesthesia protocol deviation, 1 lost to follow-up) and 2 participants in the control group (all lost to follow-up). Althoµgh these participants completed the surgery and perioperative management, they lacked follow-up outcome data including the Chalder Fatigue Scale (CFS) scores and 15-item Quality of Recovery (QoR-15) scores, resulting in missing continuous outcome data. We adopted a stratified processing strategy for different types of outcome variables as detailed below.

For continuous outcomes, including the Chalder Fatigue Scale scores, postoperative QoR-15 scores, and postoperative Visual Analogue Scale (VAS) pain scores, the analyses were performed based on observed cases who completed follow-up at the corresponding time point, with 58 participants included in the esketamine group and 58 participants in the control group for between-group comparisons. Considering the relatively small sample size of this study and the low proportion of missing data (4/120, 3.3%), methods such as multiple imputation for missing value estimation may introduce additional model assumptions and reduce the stability of the results. Therefore, no imputation was performed for continuous variables.

For the binary primary outcome (incidence of postoperative fatigue, defined as Chalder Fatigue Scale score ≥ 4), the primary confirmatory analysis was conducted in accordance with the intention-to-treat (ITT) principle, which included all 120 randomized participants (60 per group). For participants lost to follow-up with unknown fatigue status, a conservative non-responder imputation approach was applied, classifying these participants as having postoperative fatigue. A per-protocol (PP) sensitivity analysis was additionally performed, including only participants who completed all scheduled follow-up without major protocol deviations (58 per group), to evaluate the robustness of the findings under different missing data assumptions.

For binary outcomes, between-group differences were expressed as relative risk (RR), risk difference (RD), and odds ratio (OR), with corresponding 95% confidence intervals (CIs). The number needed to treat (NNT) with 95% CI was calculated for the primary binary outcome to reflect the clinical benefit of the intervention. For participants who did not complete postoperative follow-up and whose fatigue status could not be determined, a conservative non-responder imputation approach was applied, and these participants were classified as having postoperative fatigue. This approach was adopted to avoid underestimating the incidence of adverse outcomes due to loss to follow-up, thereby evaluating the robustness of the results under different missing data handling assumptions.

For other perioperative variables, including duration of surgery, anesthetic drµg consumption, safety indicators, and post-anesthesia care unit (PACU) length of stay, all data were completely recorded during the surgical procedure with no missing follow-up data. Therefore, all analyses for these variables were performed based on the full cohort of all randomized participants (60 participants per group).

In this study, appropriate analysis populations were adopted for different types of variables: completer analysis was used for continuous follow-up outcomes (58 vs. 58); ITT analysis served as the primary analysis for the binary primary outcome (60 vs. 60), with PP analysis as a sensitivity analysis; full sample analysis was applied for perioperative variables (60 vs. 60), This analytical framework was established to minimize the impact of missing data on the study conclusions while ensuring the methodological rationality of all statistical analyses. No pre-specified subgroup analyses, multivariable adjusted analyses, or exploratory post-hoc analyses were planned in the study protocol and clinical trial registration, and no such additional analyses were performed in this study, to avoid type I error inflation caused by multiple comparisons. All secondary outcome analyses were exploratory in nature and no adjustment for multiple comparisons was applied. Statistically significant findings for secondary outcomes should be interpreted as hypothesis-generating and require confirmation in future dedicated studies.

## Results

### Patients

**Fig. 1:**
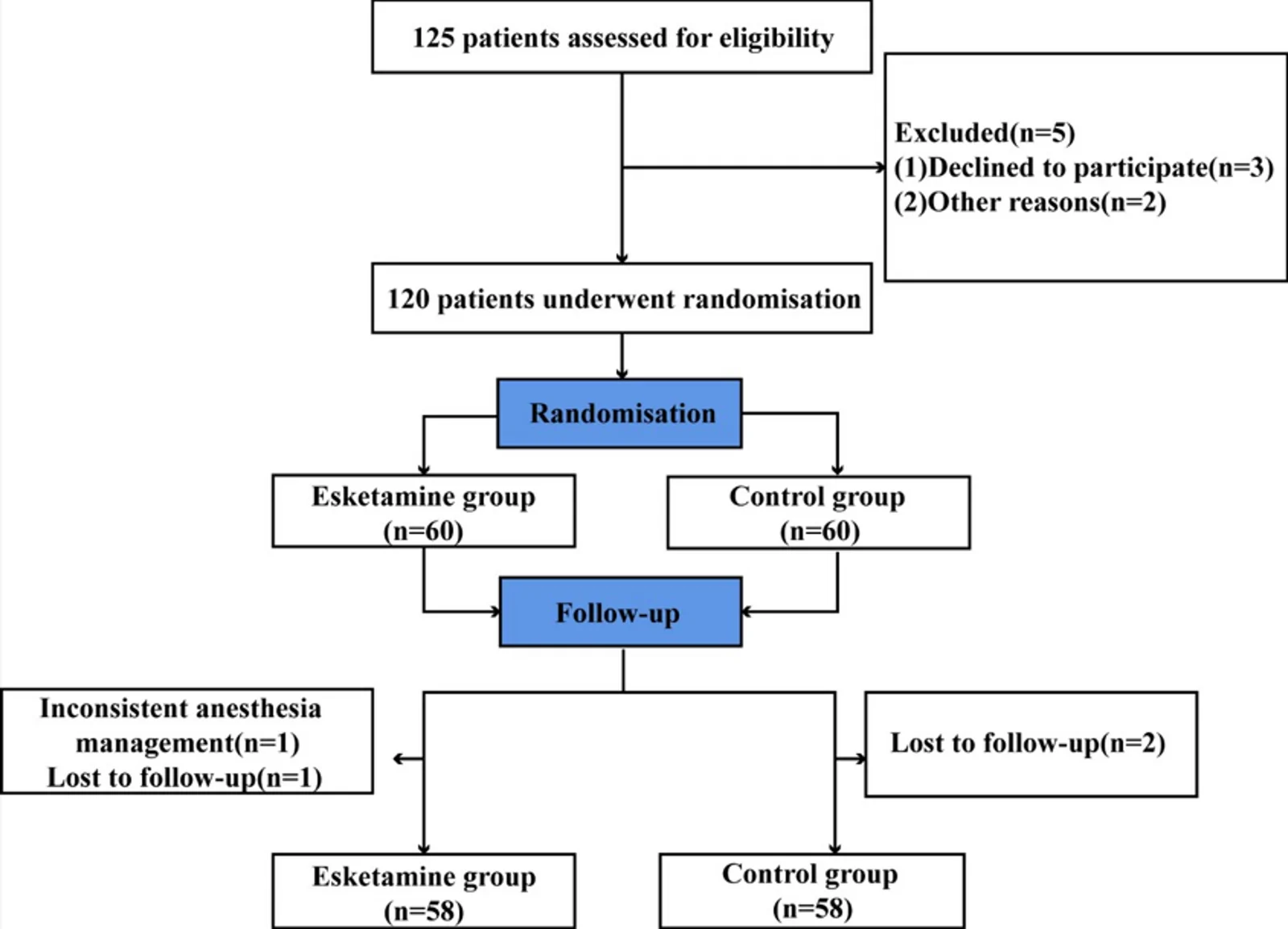
CONSORT flow diagram of this randomized, double-blind, placebo-controlled trial. This diagram illustrates the full process of patient enrollment, randomization, follow-up, and final analysis. A total of 125 patients were assessed for eligibility between November 2025 and March 2026, 5 patients were excluded before randomization (3 declined to participate, 2 excluded for other reasons), and 120 eligible patients were randomized 1:1 to the esketamine group (*n* = 60) or the normal saline control group (*n* = 60). After randomization, 1 patient in the esketamine group was excluded due to inconsistent anesthesia management, 2 patients in the esketamine group and 1 patient in the control group were lost to follow-up. Finally, 58 patients in the esketamine group and 58 patients in the control group were included in the full analysis set

Between November 2025 and March 2026, a total of 125 patients were assessed for eligibility. Of these, three declined to participate and two were excluded for other reasons: both patients were only conversant in local dialect with limited Mandarin proficiency, which impeded effective communication and prevented reliable completion of the self-reported outcome scales required by the study protocol. The final follow-up of the last enrolled participant was completed on March 11, 2026. A total of 120 eligible patients were randomized 1:1 to the esketamine group (*n* = 60) or the normal saline control group (*n* = 60). One was excluded due to inconsistent anesthesia management and three were lost to follow-up. After randomization, 58 participants in the esketamine group and 58 participants in the control group received the intended study intervention as allocated. Finally, 58 patients in the esketamine group and 58 patients in the control group were included in the primary outcome analysis and the full analysis set. Baseline characteristics were comparable between the two groups. No significant differences were observed in age, body mass index, ASA physical status, duration of surgery, type of surgery, or preoperative scores (*P* > 0.05 for all) (Fig. 1) (Table 1). The trial was officially ended after the pre-specified total sample size of 116 participants was fully enrolled and all follow-up procedures were completed. The trial was not terminated early, and all pre-specified study procedures were fully implemented as planned.

### Primary and secondary outcomes

#### Primary outcome: incidence of postoperative fatigue

For the primary outcome of the incidence of postoperative fatigue on postoperative day 1, the primary confirmatory analysis was conducted in accordance with the intention-to-treat (ITT) principle, which included all 120 randomized participants (60 per group). For participants lost to follow-up with unknown fatigue status, a conservative non-responder imputation approach was applied, classifying these participants as having postoperative fatigue.

**Fig. 2:**
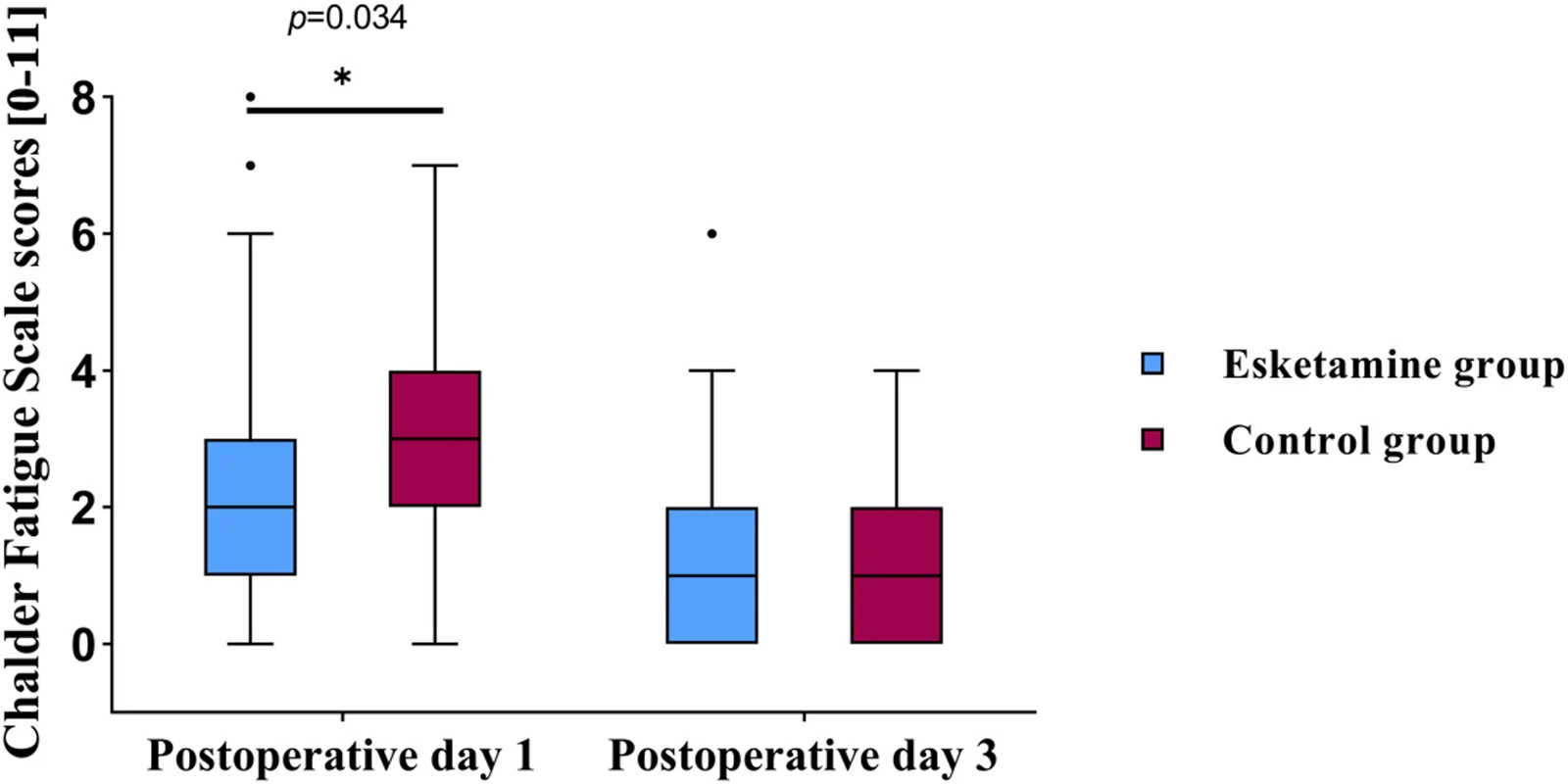
Chalder Fatigue Scale (CFS) scores of the two groups at postoperative day 1 (POD1) and postoperative day 3 (POD3). Data are presented as mean ± standard deviation. The esketamine group received continuous intraoperative infusion of 0.2 mg·kg⁻¹·h⁻¹ esketamine. The between-group difference in Chalder Fatigue Scale (CFS) scores was statistically significant at postoperative day 1 (POD1) (*P* = 0.034). For the primary binary outcome of postoperative fatigue incidence, the primary intention-to-treat analysis demonstrated a borderline statistically significant between-group difference at POD1 (*P* = 0.050). At postoperative day 3 (POD3), no statistically significant between-group difference was observed in either CFS scores or fatigue incidence (all *P* > 0.05)

**Fig. 3:**
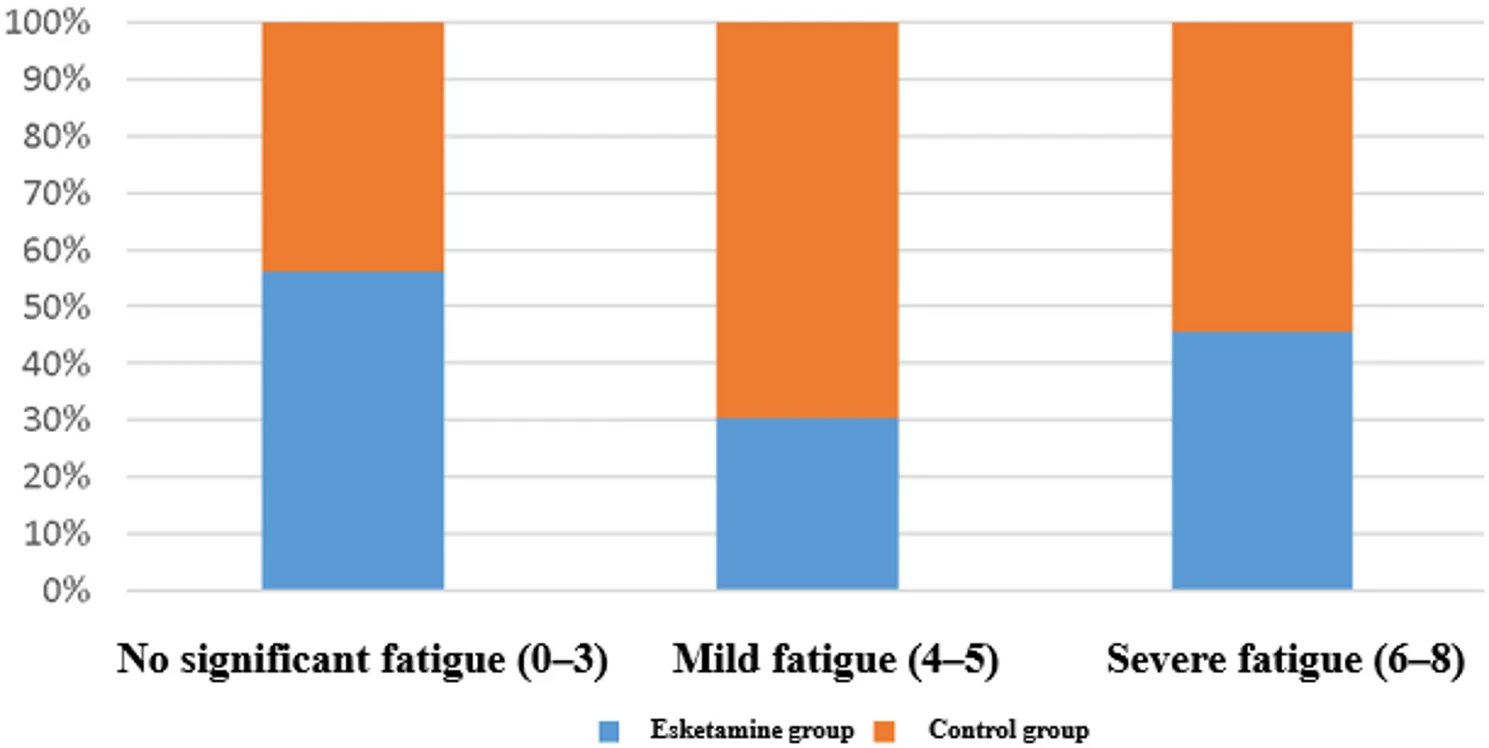
Proportion of fatigue severity grades in the two groups at postoperative day 1 (POD1). Fatigue severity was graded according to the Chalder Fatigue Scale (CFS) score: no significant fatigue (CFS score 0–3), mild fatigue (CFS score 4–5), and severe fatigue (CFS score 6–8). Postoperative fatigue was defined as a CFS score ≥ 4, which was the primary outcome of this trial. The esketamine group (*n* = 58) received continuous intraoperative infusion of 0.2 mg·kg⁻¹·h⁻¹ esketamine, and the control group (*n* = 58) received equal volume normal saline

The incidence of postoperative fatigue was 23.3% (14/60) in the esketamine group and 40.0% (24/60) in the control group (risk difference [RD] = − 16.7%, 95% CI: −33.0% to − 0.3%, relative risk [RR] = 0.58, 95% CI: 0.34 to 1.01, odds ratio [OR] = 0.46, 95% CI: 0.21 to 1.01, *P* = 0.050). The corresponding number needed to treat (NNT) was 6 (95% CI: 4 to 334) (Table 2) (Fig. 2) (Fig. 3).

**Table 2:** Chalder fatigue scale scores

Characteristic	Group E	Group C	*P* value
Chalder Fatigue Scale score on postoperative day 1(*n* = 58)	2.43 ± 1.8	3.14 ± 1.7	**0.034**
Chalder Fatigue Scale score on postoperative day 3(*n* = 58)	1.19 ± 1.2	1.28 ± 1.1	0.547
Incidence of postoperative fatigue on postoperative day 1 (*n* = 58)	20.7%	37.9%	**0.042**
Incidence of postoperative fatigue on postoperative day 1 (*n* = 60)	23.3% (14/60)	40.0% (24/60)	**0.050**

The first three items of scale score data in this table are presented based on the per-protocol (PP) completer analysis set, which includes participants who completed all scheduled follow-up assessments, with 58 patients in the esketamine group (Group E) and 58 patients in the normal saline control group (Group C). The incidence of postoperative fatigue on postoperative day 1 (POD1), the primary outcome of this trial, is presented based on the intention-to-treat (ITT) full analysis set, including all 60 randomized patients in Group E and 60 randomized patients in Group C. Data are presented as mean ± standard deviation (SD) for scale scores, and n (%) for the incidence of postoperative fatigue. Group E received continuous intraoperative infusion of 0.2 mg·kg⁻¹·h⁻¹ esketamine; Group C received equal volume of normal saline. Postoperative fatigue was defined as a Chalder Fatigue Scale score ≥ 4. POD3: postoperative day 3. A *P* value < 0.05 was considered statistically significant

Values in bold indicate statistically significant between-group differences (*P* <0.05)

A per-protocol (PP) sensitivity analysis was performed including only participants who completed all scheduled follow-up without major protocol deviations (58 per group), to evaluate the robustness of the findings under different missing data assumptions. The PP analysis showed consistent results with a larger effect size: the incidence of postoperative fatigue was 20.7% in the esketamine group and 37.9% in the control group (OR = 0.43, 95% CI 0.19–0.98, *P* = 0.042).

#### Secondary outcomes: fatigue scores, quality of recovery, and pain scores

**Fig. 4:**
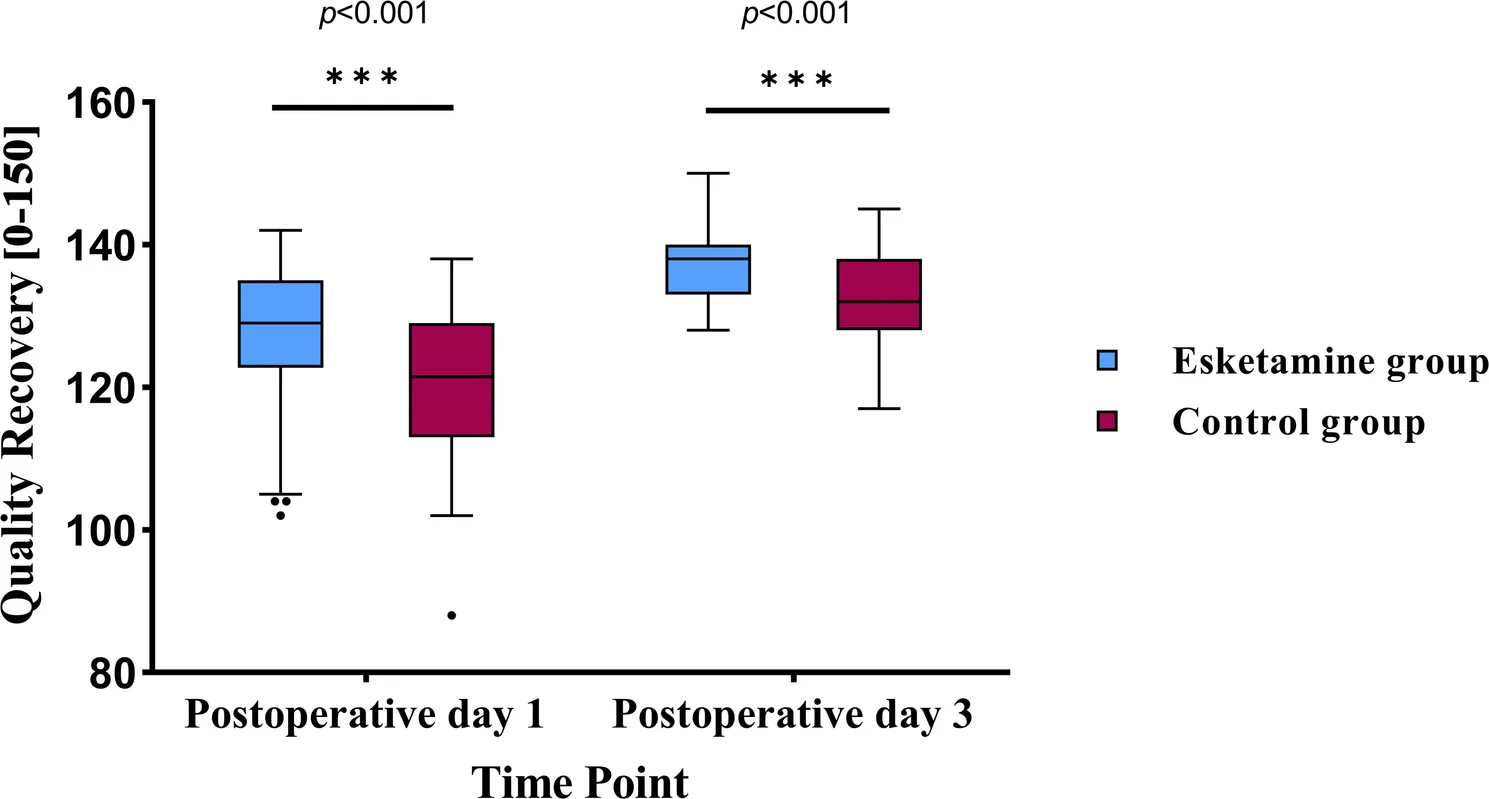
15-item Quality of Recovery (QoR-15) scores of the two groups at postoperative day 1 (POD1) and postoperative day 3 (POD3). Data are presented as mean ± standard deviation. The esketamine group received continuous intraoperative infusion of 0.2 mg·kg⁻¹·h⁻¹ esketamine, and the control group received equal volume normal saline (*n* = 58 per group). A higher QoR-15 score indicates better postoperative recovery quality. The QoR-15 scores of the esketamine group were significantly higher than those of the control group at both POD1 and POD3, with a statistically significant between-group difference at both time points (all *P* < 0.001)

**Fig. 5:**
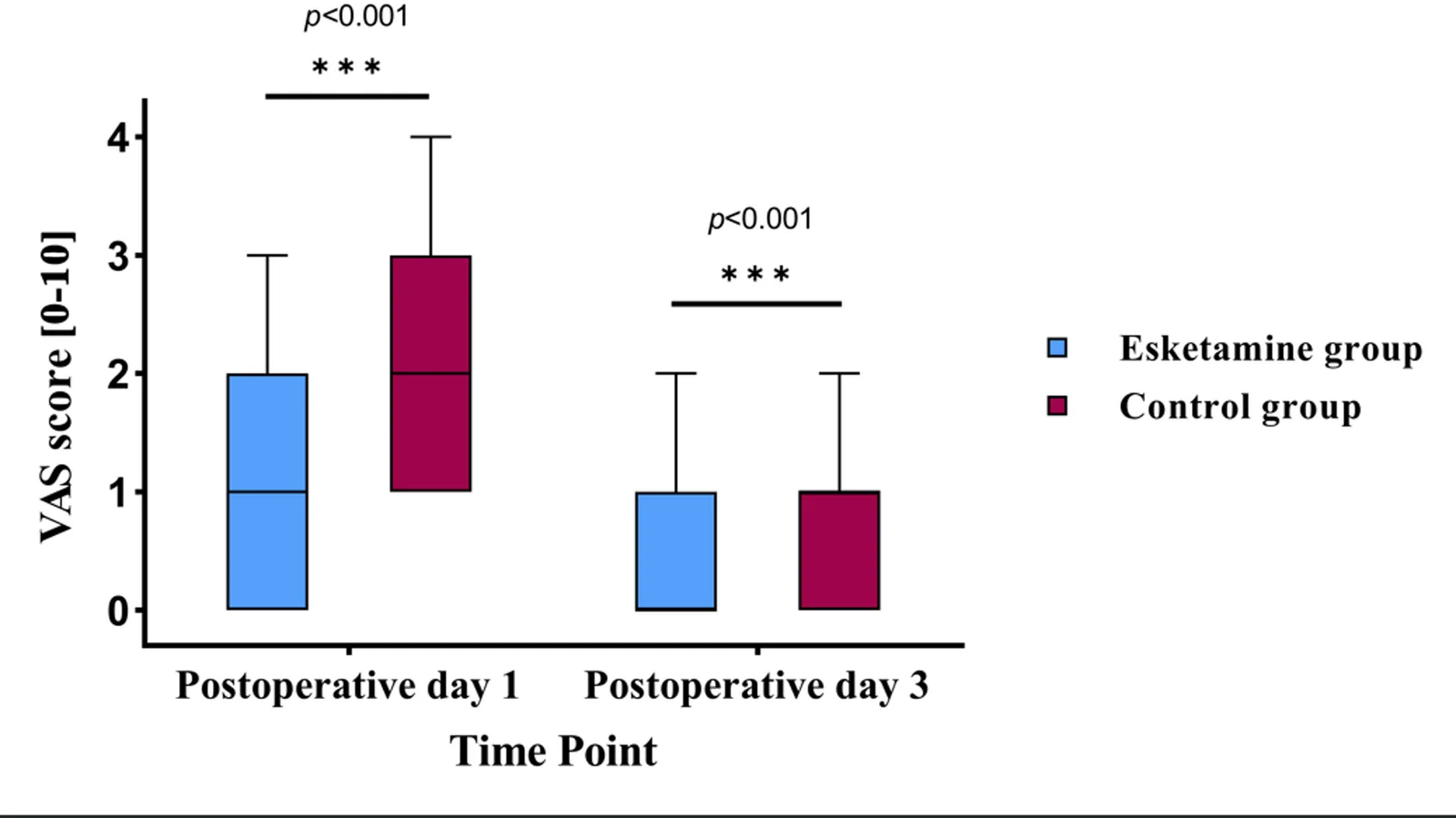
Visual Analogue Scale (VAS) pain scores of the two groups at postoperative day 1 (POD1) and postoperative day 3 (POD3). Data are presented as mean ± standard deviation. The esketamine group received continuous intraoperative infusion of 0.2 mg·kg⁻¹·h⁻¹esketamine, and the control group received equal volume normal saline (*n* = 58 per group). The VAS score ranges from 0 to 10, where 0 = no pain and 10 = worst possible pain. The VAS pain scores of the esketamine group were significantly lower than those of the control group at both POD1 and POD3, with a statistically significant between-group difference at both time points (all *P* < 0.001)

For secondary outcomes, the esketamine group had significantly lower Chalder Fatigue Scale scores (2.43 ± 1.8 vs. 3.14 ± 1.7, *P* = 0.034), lower Visual Analogue Scale (VAS) pain scores (1.1 ± 0.9 vs. 2.0 ± 0.8, *P* < 0.001), and higher 15-item Quality of Recovery (QoR-15) scores (127.2 ± 9.3 vs. 121.0 ± 10.3, *P* < 0.001) on postoperative day 1 compared with the control group (Table 3) (Fig. 4) (Fig. 5). On postoperative day 3, there was no statistically significant difference in Chalder Fatigue Scale scores between the two groups (*P* > 0.05), while statistically significant differences were observed in QoR-15 scores and VAS pain scores (all *P* < 0.001).

**Table 3:** Quality of recovery score

Characteristic	Group E (*n* = 58)	Group C (*n* = 58)	Mean Difference (95% CI)	*P* value
QoR-15 score on postoperative day 1	127.2 ± 9.3	121.0 ± 10.3	6.2(2.6, 9.8)	**< 0.001**
QoR-15 score on postoperative day 3	136.5 ± 4.8	132.7 ± 6.1	3.8(1.8, 5.8)	**< 0.001**
VAS score on postoperative day 1	1.07 ± 0.9	2.0 ± 0.8	−0.93(−1.24, − 0.62)	**< 0.001**
VAS score on postoperative day 3	0.31 ± 0.5	0.72 ± 0.6	−0.41(−0.61, − 0.21)	**< 0.001**

Data are presented as mean ± standard deviation (SD). Group E: esketamine group; Group C: control group (*n* = 58 per group). QoR-15: 15-item Quality of Recovery scale, a higher score indicates better postoperative recovery quality; VAS: Visual Analogue Scale, score ranges from 0 (no pain) to 10 (worst possible pain). A *P* value < 0.05 was considered statistically significant

Values in bold indicate statistically significant between-group differences (*P* < 0.05)

#### Secondary outcomes: perioperative drug consumption

Intraoperative sufentanil consumption was lower in the esketamine group (20.0 [20,25] µg vs. 25.0 [20,30] µg, *P* = 0.006), as was ephedrine consumption (4.00 ± 1.5 mg vs. 5.92 ± 3.04 mg, *P* = 0.012), while remifentanil consumption did not differ significantly between the groups (*P* > 0.05). No significant differences were observed between the two groups in extubation time or PACU stay duration or rescue analgesia rate (*P* > 0.05) (Table 4) (Table 5).

**Table 4:** Surgical characteristics

Characteristic	Group E (*n*=60)	Group C (*n*=60)	*P* value
Surgical data, median (interquartile range)			
Duration of surgery, min	119.0(90.8,150)	124.5(99.8,153)	0.479
Extubation time, min	21.5(15,36)	24(15,34)	0.962
PACU stay duration, min	49.0(39,64)	54.0(34,64)	0.964
Sevoflurane consumption, mL	24.6(17.7,34.1)	24.7(16.9,32.6)	0.846
Sufentanil consumption, μg	20.0(20,25)	25.0(20,30)	**0.006**
Remifentanil consumption, mg	1.24(0.9,1.6)	1.23(0.9,1.57)	0.813
Type of surgery, n (%)			
Ovarian lesion resection:	31, (52%)	28, (47%)	0.715
Uterine lesion resection:	11, (18%)	16, (27%)	0.382
Tubal surgery:	46, (77%)	40, (67%)	0.311
Total hysterectomy:	23, (38%)	17, (28%)	0.333
Hysteroscopic surgery:	16, (27%)	23. (38%)	0.242
Other:	0, (0%)	2, (3%)	0.496
Vasoactive agents, mean ± SD, mg			
Ephedrine	4.00±1.5	5.92±3.04	**0.012**
Atropine	0.4±0.13	0.42±0.11	0.664

All data in this table are presented based on the intention-to-treat (ITT) full analysis set, with 60 patients in the esketamine group (Group E) and 60 patients in the normal saline control group (Group C). Data are presented as n (%), mean ± standard deviation (SD), or median (interquartile range, IQR). Group E received continuous intraoperative infusion of 0.2 mg·kg⁻¹·h⁻¹ esketamine; Group C received equal volume of normal saline. PACU: post-anesthesia care unit. A *P* value < 0.05 was considered statistically significant

*Patients may have undergone multiple concurrent surgical procedures during the same operation, therefore the percentages of each surgical type are not mutually exclusive and do not sum to 100%

Values in bold indicate statistically significant between-group differences (*P* < 0.05)

**Table 5:** Safety indicators

Adverse event, *n* (%)	Group E (*n* = 58)	Group C (*n* = 58)	*P* value
Nausea and vomiting	6(10%)	9(15%)	0.58
Hypoxaemia	1(1.7%)	0(0)	1
Hypotension	0(0)	0(0)	1
Hypertension	1(1.7%)	2(3.3%)	1
Postoperative agitation	2(3.3%)	0(0)	0.49
Delayed emergence	1(1.7%)	0(0)	1
Rescue analgesia	5(8.3%)	13(21.7%)	0.071
Hallucinations, n (%)	0(0)	0(0)	1
Nightmares, n (%)	0(0)	0(0)	1

Note: All data in this table are presented based on the intention-to-treat (ITT) full analysis set, with 60 patients in the esketamine group (Group E) and 60 patients in the normal saline control group (Group C). Data are presented as n (%). Group E received continuous intraoperative infusion of 0.2 mg·kg⁻¹·h⁻¹ esketamine; Group C received equal volume of normal saline. Rescue analgesia was defined as intravenous administration of 100 mg tramadol for a Visual Analogue Scale (VAS) pain score ≥ 4. A *P* value < 0.05 was considered statistically significant. No severe adverse events occurred in either group during the study period

### Side effects and adverse events

No significant differences were observed between the two groups in the incidence of adverse reactions, including nausea and vomiting, hypoxaemia, hypertension, postoperative agitation, or delayed emergence (*P* > 0.05 for all). The incidence of nausea and vomiting was 10% in the esketamine group and 15% in the control group. No severe adverse events occurred in either group (Table 5).

## Discussion

The results of our study indicate that intraoperative infusion of low-dose esketamine (0.2 mg·kg⁻¹·h⁻¹) is associated with a reduced incidence of postoperative fatigue on day 1 in patients undergoing gynecological laparoscopic surgery. In the primary ITT analysis, the incidence decreased from 40.0% in the control group to 23.3% in the esketamine group, with a borderline statistically significant difference (*P* = 0.050). The per-protocol sensitivity analysis confirmed a statistically significant reduction, supporting the robustness of the finding. These findings are generally consistent with existing literature. A study in patients undergoing thyroid surgery showed that intravenous esketamine 0.5 mg/kg followed by continuous infusion at 4 µg·kg⁻¹·min⁻¹ improved postoperative fatigue and reduced the incidence of postoperative fatigue syndrome on day 1 [30]. This trial provides preliminary evidence supporting the anti-fatigue effect of esketamine on POD1 in patients undergoing gynecological laparoscopic surgery, supplementing evidence for its application in abdominal surgery. Current studies covering various surgical types, including thyroid surgery, colorectal cancer surgery, and radical gastrectomy, have all observed the ameliorative effect of esketamine on postoperative fatigue, sµggesting that the effect may be generalizable rather than limited to specific surgical procedures [31, 32]. 

In the present study, the primary ITT analysis including all randomized participants showed a borderline significant reduction in postoperative fatigue incidence in the esketamine group, with a conservative non-responder imputation approach applied for missing follow-up data. The per-protocol sensitivity analysis, which included only participants who completed all follow-up without protocol deviations, showed a consistent direction of effect with a larger effect size and reached conventional statistical significance. Although the primary ITT analysis reached a borderline *P* value of 0.050, the direction of the effect was consistent across both analytical strategies, and the absolute risk reduction of 16.7% still presents clear clinical relevance. The statistically significant result in the PP sensitivity analysis further supports that esketamine has a genuine beneficial effect on reducing early postoperative fatigue.

A total of 4 participants in this study did not complete postoperative follow-up, and their postoperative fatigue status could not be directly determined. In the ITT sensitivity analysis, these participants were uniformly classified as having postoperative fatigue, which increased the number of events in both groups. This approach is based on a conservative statistical assumption that tends to underestimate the intervention effect estimate, thus diluting the between-group difference to a certain extent. In addition, the overall loss to follow-up rate was low (3.3%, 4/120), with a balanced distribution between the two groups (2 cases in each group), indicating that the impact of missing data on the overall results was limited.

Althoµgh the ITT primary analysis did not reach the conventional threshold of statistical significance (*P* = 0.050), the direction of the effect was consistent across both ITT and PP analyses, and the absolute risk reduction of 16.7% corresponds to a number needed to treat (NNT) of 6. This effect size is clinically meaningful and comparable to standard perioperative interventions: for reference, the NNT for multimodal analgesia to reduce moderate-severe postoperative pain is 4–7, and the NNT for prophylactic antiemetics is 5–8.

While the 95% CI for risk difference (− 33.0% to − 0.3%) is relatively wide, it does not cross the null value for harm, and the lower bound indicates a potentially large clinical benefit. The consistent direction of effect across both analytical strategies supports the robustness of the findings.

The results of this study showed a certain degree of consistency under different analytical strategies, sµggesting that the study conclusions have acceptable robustness. However, considering the relatively limited sample size and the impact of the missing data handling approach, the intervention effect of esketamine on postoperative fatigue still needs to be further verified in larger-sample clinical trials.

In our study, the Chalder Fatigue Scale score on postoperative day 1 was significantly lower in the esketamine group than in the control group, but no significant difference was observed between the two groups on postoperative day 3. This finding sµggests that the effect of esketamine on postoperative fatigue is time-dependent [33]. Esketamine reduced the Chalder Fatigue Scale score on postoperative day 1, but this advantage disappeared by postoperative day 3. This can be explained by the self-limiting nature of postoperative fatigue—as fatigue symptoms naturally diminish over time in the control group, by postoperative day 3, the fatigue levels in the control group may have become comparable to those in the esketamine group, resulting in the disappearance of the intergroup difference. Nevertheless, the first 24 h after surgery are a critical period for emergence from anesthesia and initiation of early mobilization [34]. Alleviating fatigue during this period facilitates earlier ambulation and more active participation in rehabilitation, highlighting the clinical value of early intervention with esketamine.

The results of our study demonstrate that continuous infusion of esketamine significantly improves quality of recovery on both postoperative day 1 and postoperative day 3, findings consistent with those observed in a previous study of total laparoscopic hysterectomy [10]. Analysis of primary indicators revealed that the effects of esketamine on different outcomes varied over time. Improvement in fatigue was observed only up to postoperative day 1, whereas improvements in quality of recovery scores and pain scores persisted up to postoperative day 3. This may be attributed to differences in the evaluation dimensions of the scales. The Chalder Fatigue Scale primarily focuses on fatigue symptoms, whereas the Quality of Recovery scale encompasses multiple aspects including physical comfort, emotional state, and daily activities [35]. The 6.2-point difference in QoR-15 scores on POD1 exceeds the minimal clinically important difference (MCID) of 5 points for the QoR-15 scale, confirming a clinically meaningful improvement in recovery quality. This sµggests that esketamine may promote early active participation in rehabilitation throµgh multiple mechanisms, and the functional improvements resulting from early mobilization may persist after the pharmacological effects subside, leading to sustained improvement in overall postoperative recovery quality.

In our study, Visual Analogue Scale (VAS) pain scores on both postoperative day 1 and postoperative day 3 were significantly different compared with the control group. This sµggests that esketamine may provide sustained analgesic effects, possibly related to its mechanisms of inhibiting central sensitization and preventing hyperalgesia [36, 37]. Sustained analgesia in turn creates favorable conditions for early mobilization and rehabilitation training. Furthermore, althoµgh there was no significant difference in intraoperative remifentanil consumption between the two groups, sufentanil consumption differed significantly, indicating that esketamine provides partial analgesic coverage throµgh NMDA receptor antagonism, thereby reducing dependence on long-acting opioids. Notably, althoµgh reduced sufentanil consumption might theoretically lead to inadequate postoperative analgesia, pain scores were actually lower in the esketamine group, further confirming the analgesic effect of esketamine. The reduction in sufentanil consumption also implies a corresponding decrease in patient exposure to opioid-related adverse effects.

The present study showed statistically significant between-group differences in postoperative Visual Analogue Scale (VAS) pain scores on postoperative day 1 and day 3, with lower pain scores observed in the esketamine group. However, there was no significant difference in the rescue analgesia rate between the two groups. As postoperative pain is widely recognized as a major contributing factor to postoperative fatigue, these findings confirm that the beneficial effect of esketamine on postoperative fatigue was not caused by insufficient analgesia in either group under the current study protocol.

It should be noted that all patients in both groups received standardized bilateral transversus abdominis plane (TAP) block at the end of surgery. This uniform multimodal analgesic regimen may have reduced overall pain severity across both groups, potentially attenuating the observed between-group difference in pain scores. The fact that a significant difference in pain scores was still observed despite TAP blockade indicates that esketamine provides additional analgesic benefit beyond the TAP block alone.

Postoperative fatigue is closely interlinked with pain, sleep disturbances, and depressive symptoms. Esketamine has beneficial effects on all these domains, so the observed anti-fatigue effect may be indirect, mediated by improvements in these intermediate factors. Our study only measured pain and overall recovery quality, not sleep architecture or depressive symptoms.

The results of our study also showed that ephedrine consumption was significantly different between the esketamine group and the control group, consistent with the pharmacological property of esketamine that activates the sympathetic nervous system and promotes catecholamine release, thereby maintaining more stable haemodynamic status and indirectly contributing to overall recovery.

Regarding other safety indicators, the esketamine group had 6 cases of nausea and vomiting, 1 case of hypoxaemia, 1 case of hypertension, 2 cases of postoperative agitation, and 1 case of delayed emergence, while the control group had 9 cases of nausea and vomiting and 2 cases of hypertension, all within expected ranges. In our study, the low-dose regimen (0.2 mg·kg⁻¹·h⁻¹) resulted in only 1 case of delayed emergence, a low incidence that supports the safety of the low-dose regimen.

Esketamine-specific psychiatric adverse reactions, such as hallucinations, nightmares, and diplopia, were not detected in our study. This may be related to two factors. First, patients with a history of psychiatric disorders or psychiatric medication use were excluded from our study. Second, the continuous infusion regimen used in our study avoided peak plasma concentrations, reducing the risk of psychiatric side effects.

### Limitations

Several limitations of this randomized controlled trial should be acknowledged. This study adopted relatively broad inclusion criteria, and strictly followed the pre-specified exclusion criteria to enroll patients undergoing various eligible gynecological laparoscopic procedures, including hysterectomy, adnexal surgery, and other common gynecological laparoscopic operations. However, there are inherent differences in surgical trauma, operation duration, and the magnitude of systemic stress response among different surgical procedures. We did not perform in-depth subgroup analyses stratified by operation duration or specific surgical type in the present study, and further subgroup analyses are warranted in future studies to explore the differential efficacy of esketamine across different gynecological laparoscopic procedures.

There are limitations in the dose design of this study. Prior to the initiation of this prospective randomized controlled trial, we conducted a retrospective statistical analysis of clinical data collected in our institution, which did not involve any prospective human intervention. The retrospective analysis revealed that patients receiving continuous infusion of high-dose esketamine (0.3 mg·kg⁻¹·h⁻¹) had a higher incidence of postoperative agitation in the post-anesthesia care unit (PACU) than those receiving low-dose regimens. Based on safety and ethical considerations, only the low-dose esketamine regimen (0.2 mg·kg⁻¹·h⁻¹) was adopted in this formal prospective trial. Our study did not set up multiple dose groups to directly compare the efficacy of high-dose versus low-dose esketamine in improving postoperative fatigue and quality of recovery, and further multi-arm parallel controlled studies are needed to explore and verify the optimal dose of esketamine for this indication.

The follow-up period of this study was mainly limited to 72 h after surgery, and the long-term efficacy of intraoperative low-dose esketamine remains to be verified by subsequent studies. Since a proportion of patients were discharged within 72 h after surgery, we only observed early postoperative indicators in the present study. Postoperative fatigue can persist for weeks or even longer after surgery; existing studies have shown that the ameliorative effect of esketamine on fatigue can be sustained until 7 days after surgery, but long-term follow-up data are still lacking. This study mainly focused on early postoperative fatigue, and the incidence, duration of postoperative fatigue in the medium and long term (e.g., 1 month and 3 months after surgery), as well as its impact on patients’ long-term quality of life, need to be further clarified. In subsequent studies, we plan to further extend the time window of postoperative follow-up to comprehensively collect the incidence of postoperative complications and long-term postoperative fatigue, so as more fully evaluate the long-term prognosis of patients.

This study focused exclusively on clinical outcomes, and all mechanistic interpretations are hypothesis-generating and not directly tested in this trial. While we have drawn on existing literature to propose potential pathways (including anti-inflammatory effects, central sensitization inhibition, and monoaminergic modulation), these mechanisms were not assessed via biomarker measurement in our study. Specifically, we did not measure perioperative levels of interleukin-6 (IL-6), tumor necrosis factor-α (TNF-α), or brain-derived neurotrophic factor (BDNF), so we cannot confirm the exact molecular pathways throµgh which esketamine may reduce postoperative fatigue. Further studies with serial biomarker sampling are required to elucidate the underlying biological mechanisms.

While the difference in ephedrine consumption was statistically significant, the absolute difference was small (1.56 mg) and not clinically relevant, as all patients maintained hemodynamic stability within target ranges.

Specifically, we did not measure the levels of inflammatory indicators including interleukin-6 (IL-6), tumor necrosis factor-α (TNF-α), and brain-derived neurotrophic factor (BDNF) during the perioperative period, which prevents us from clarifying the specific signaling pathways throµgh which esketamine alleviates postoperative fatigue at the molecular level. Further studies with serial measurements of these relevant biomarkers are warranted to deeply explore the intrinsic biological mechanism of esketamine in improving postoperative fatigue.

Although we preoperatively excluded patients with a history of psychiatric disorders or psychotropic medication use, and esketamine-related psychiatric adverse reactions are mostly transient and can resolve spontaneously within 15 to 60 min after drµg discontinuation, this study still lacks a specialized assessment of postoperative psychiatric-related adverse events. Relevant events were only reflected in the emotion-related items of the 15-item Quality of Recovery (QoR-15) scale, and were not systematically analyzed as independent observation indicators. Specifically, we did not systematically assess esketamine-class neuropsychiatric adverse events including hallucinations, nightmares, diplopia, and dissociation symptoms. Relevant events were only indirectly reflected in the emotional items of the QoR-15 scale, and were not analyzed as independent safety endpoints. We did not systematically assess preoperative psychological status (anxiety, depression, fear of surgery), which are known risk factors for postoperative fatigue. Subclinical psychological distress may have influenced our results.

Future studies should adopt validated specialized psychiatric assessment tools, with follow-up staff trained in psychometric evaluation, to systematically document neuropsychiatric adverse events including anxiety, depression, hallucinations, and nightmares.

### Generalisability and clinical applicability

The findings of this trial are generalisable to adult female patients aged 18–65 years with ASA physical status I–II, undergoing elective gynecological laparoscopic surgery in tertiary hospitals in China. The study population aligns with most patients receiving routine gynecological laparoscopic surgery in clinical practice, and the low-dose esketamine infusion regimen is easy to implement, low-cost, and has a manageable safety profile, supporting its uptake in routine clinical anesthesia practice.

However, several factors limit broader extrapolation. First, patients with ASA physical status III or higher were excluded; these patients typically have higher baseline fatigue risk and may respond differently to esketamine. Second, this was a single-center academic hospital study, and results may not generalise to community hospitals or regions with different clinical practice patterns. Third, our findings cannot be directly extended to other surgical populations such as major abdominal, orthopedic, or cardiac surgery, which involve greater surgical trauma and higher baseline fatigue rates. Fourth, we tested only one specific dose and infusion regimen; results may not apply to bolus dosing, postoperative infusion, or different dose levels.

Multicenter trials including broader patient populations and surgical types are needed to confirm the generalisability of these findings.

## Conclusion

Low-dose esketamine (0.2 mg·kg⁻¹·h⁻¹) alleviates postoperative fatigue, reduces its incidence, improves quality of recovery, and provides effective postoperative analgesia in patients undergoing gynecological laparoscopic surgery. It also reduces perioperative opioid and vasoactive agent requirements without increasing the incidence of related adverse reactions.

## Data Availability

The raw data generated and analyzed during this study are not publicly available due to the protection of participants’ privacy and sensitive clinical data. The datasets are available from the corresponding author upon reasonable academic request, and the request will be reviewed and approved by the ethics committee of the study institution before data sharing to ensure compliance with ethical requirements for privacy protection.
